# Foreign Correspondence

**Published:** 1869-12

**Authors:** 


### Foreign Correspondence.

R. R. ALLGEMEINEN KRAUKENHAUSE,
VIENNA, *October 30, 1869.*

DEAR EXAMINER:—As has been the case in many instances, it was vacation when I visited Prag, and I was unable to learn many particulars about medical matters. The hospital has about 800 beds, and presents about the same opportunities as Vienna for studying obstetrics and diseases of children. The number of cases is smaller, but there is only about one-fourth the number of students (300).

Prof. Scanzoni and Tröltsch, at Wurtzburg, attract many admirers. I have heard that one of Scanzoni's assistants is an American.

A reading room has been opened in the hospital, which is supplied with about fifty of the leading medical journals of the continent. The *London Lancet*, and *New York Medical Record* are the only two English journals. Several American journals will be added after the first of January. The rooms are open from 10 A.M. to 11 P.M. The terms of admission are 60 cts. per month.

The hospital here has undergone some changes since I last wrote from here. Many of the wards and rooms have been freshly painted, grained, frescoed, etc., while the whole building has been thoroughly cleaned outside and inside, and presents a much finer appearance than when I went away. Several masons have been at work enlarging the windows, and adding numerous ventilators, which has greatly improved the air in the wards.

Profs. Hebra and Sigmund have recently been made Professors Ordinary, and Prof. Zeisl Primarius Mauthner, formerly Assistant of Prof. Arlt, and one of the best eye teachers in Germany, has been appointed Professor at Innsbruck. There are about thirty American students here, two of whom are ladies. The cost of many private courses is somewhat greater than last year.

I think I will close this letter by a brief reference to tumor cavernosis, as several cases have recently fallen under my observation, and as the best method of treatment still stands in discussion.

Telangiektasie is another species of tumor, which, with the one above mentioned, are embraced under the common name of angiome. By this term angiome, we understand a tumor composed of vessels bound together by connective tissue. Examined microscopically, they present a picture similar to that of the corpus cavernosum of the penis: hence they are often called erectile tumors, as they increase or decrease in size according as they are or are not filled with blood.

Several theories have been advanced as to the manner of development of the tumor cavernosus. For example, one author supposes that the venous capillaries and small veins become enlarged or varicose, until their walls press against each other and become gradually absorbed. Another, that small caverns are formed, first in the connective tissue, and become later, in connexion with the vessels, and that perhaps new blood corpuscles were formed from the cells of the connective tissue, while yet another maintains that the calibre of the vessels is enlarged by the contraction (cicactrical) of connective tissue. Many other similar suppositions might be added. The internal wall of these caverns is generally paved with spindle-shaped cells. Many of these theories would lead us to suppose that the cavernous tumor is the result of inflammation, yet we seldom observe any signs of inflammation in the course of the disease, if I may so call it, and perhaps yet seldomer discover any signs of this process with the microscope. The diagnosis of these tumors is not always easy, as they are often of a fluctuating feel, and can be mistaken for cysts, abscesses, aneurisms, lipomas, etc.; also, with telangiektosii, when this is not superficial. They ordinarily occur in the subcutaneous tissue, but frequently embrace the muscles, and I think Prof. Schuh states in his pathology that they frequently are developed in the bones, but later writers say this occurs very seldom. I have also seen them in the liver and spleen, and they occur in the kidneys. Of seven cases that I have chanced to see in the last two months, the tumor was situated on the upper lip in two cases, at the inf. angle of the scapula in two cases, and of the other three, one was on the temporal region, another on the cheek, another just above the knee. All were on children, under five years of age, and four on infants.

Prof. Heine, of Heidelberg, has lately written quite an extensive article on "Angioma Arterialle Racemosum." A large per cent. of his reported cases was situated about the head, with a prediliction for the ear.

As is well known, zelangiektain, or mother-mark, is generally hereditary, and ordinarily the tumor cavernous develops itself in early youth, and may be said also to be hereditary, or the result of a similar diathesis, as they extremely seldom occur in later life, when we most generally see other varicose conditions. As above mentioned, telangiektosii generally affects the superficial, sometimes the deeper tissues. Its course is slow and painless—commonly comes to a standstill—sometimes continues to increase. Billroth saw such a tumor as the first on a boy five years old. The tumor cavernosus is seldom congenital. Its occurs most frequently on the face and extremities—less frequently on the body. There is sometimes weakness of the muscles of, or near the part affected, and frequently pain in the region. The tumor cavernosus may become dangerous to life when embracing the muscles and bones. It sometimes becomes greatly reduced in size by thrombus, but a case of complete spontaneous obliteration I am unable to find record of. Various modes of treatment have been tried, such as compression, ligation of the arteries, injection of various substances, removal by knife, galvano caustic, etc. The first two methods are fruitless in most cases. The galvano caustic is a favorite method with Prof. Billroth at the present time. About a year ago, I saw a patient three years old, in his wards, with a tumor about as large as a hen's egg on the upper lip. Repeated injections of carbolic acid, tinct. iodine, liquor ferri sesqui chloridi, etc., were of no avail. The tumor meantime continued to increase in size. Finally, a wire, armed with a needle, was drawn through the tumor, and the suds attached to the battery. The tumor was thus transfixed in various directions by the wire at white heat. The tumor decreased in size rapidly, and, after one or two repetitions of the process, the child was discharged cured. Removal by knife would have necessitated a plastic and consequent deformity of the face. In other cases, it is sufficient to puncture the tumor in several places with the caustic needle. While in other cases the whole surface of the tumor may be cauterized, and so soon as the slough becomes detached, the process may be repeated, if any traces of the tumor remains. The wound is treated as an ordinary ulcer. By this method there is little or no fear of hemorrhage, which is often a troublesome complication when these tumors are removed with the knife. I may also add, that the galvano caustic is extensively used here in amputating the neck of the uterus in prolapsus, amputating the penis, cauterizing ulcerating epitheliomas, removing polypous tumors, and for other similar operations. Yours, truly, F.

